# Modelling Long Term Disability following Injury: Comparison of Three Approaches for Handling Multiple Injuries

**DOI:** 10.1371/journal.pone.0025862

**Published:** 2011-09-30

**Authors:** Belinda J. Gabbe, James E. Harrison, Ronan A. Lyons, Damien Jolley

**Affiliations:** 1 Department of Epidemiology and Preventive Medicine, Monash University, The Alfred, Melbourne, Victoria, Australia; 2 National Trauma Research Institute, The Alfred, Melbourne, Victoria, Australia; 3 Research Centre for Injury Studies, Flinders University, Adelaide, South Australia, Australia; 4 College of Medicine, Swansea University, Singleton Park, Swansea, United Kingdom; University of Otago, New Zealand

## Abstract

**Background:**

Injury is a leading cause of the global burden of disease (GBD). Estimates of non-fatal injury burden have been limited by a paucity of empirical outcomes data. This study aimed to (i) establish the 12-month disability associated with each GBD 2010 injury health state, and (ii) compare approaches to modelling the impact of multiple injury health states on disability as measured by the Glasgow Outcome Scale – Extended (GOS-E).

**Methods:**

12-month functional outcomes for 11,337 survivors to hospital discharge were drawn from the Victorian State Trauma Registry and the Victorian Orthopaedic Trauma Outcomes Registry. ICD-10 diagnosis codes were mapped to the GBD 2010 injury health states. Cases with a GOS-E score >6 were defined as “recovered.” A split dataset approach was used. Cases were randomly assigned to development or test datasets. Probability of recovery for each health state was calculated using the development dataset. Three logistic regression models were evaluated: a) additive, multivariable; b) “worst injury;” and c) multiplicative. Models were adjusted for age and comorbidity and investigated for discrimination and calibration.

**Findings:**

A single injury health state was recorded for 46% of cases (1–16 health states per case). The additive (C-statistic 0.70, 95% CI: 0.69, 0.71) and “worst injury” (C-statistic 0.70; 95% CI: 0.68, 0.71) models demonstrated higher discrimination than the multiplicative (C-statistic 0.68; 95% CI: 0.67, 0.70) model. The additive and “worst injury” models demonstrated acceptable calibration.

**Conclusions:**

The majority of patients survived with persisting disability at 12-months, highlighting the importance of improving estimates of non-fatal injury burden. Additive and “worst” injury models performed similarly. GBD 2010 injury states were moderately predictive of recovery 1-year post-injury. Further evaluation using additional measures of health status and functioning and comparison with the GBD 2010 disability weights will be needed to optimise injury states for future GBD studies.

## Introduction

The Global Burden of Disease (GBD) Study estimated the burden of injury based on selected injury health states [1], [2]. The injury health state can represent a specific injury (e.g. fractured neck of femur) or a group of injuries (e.g. fractured humerus, scapula or clavicle). A disability weight, and an estimated duration of disability, were assigned to each injury health state, and then combined with incidence or prevalence data for the health state to calculate the associated Years Lived with Disability (YLD) component of the Disability Adjusted Life Years (DALY), the metric commonly used to calculate burden [1], [3]. Hospitalisations data were predominantly used to establish the incidence of the injury health states and the principal (or first listed) diagnosis was mapped to the injury health states for application of the disability weight and duration, and calculation of YLDs.

Limitations to the GBD Study methodology have been identified. Firstly, the number of health states was limited to 33, and the extent to which these combine injuries with different disability outcomes into a single injury health state was not evaluated. Secondly, durations of disability were derived from expert opinion, and disability weights from panel studies, rather than empirical data questioning the validity of these key elements of the YLD calculations. Thirdly, the approach ignored the potential impact of multiple injuries on disability estimates. The global burden of disease estimates are being updated in the GBD 2010 Study [4] and the Injury Expert Group (GBD-IEG) was established to address the GBD study methodology used to estimate burden of injury (http://sites.google.com/site/gbdinjuryexpertgroup/). This group contributed to the revision of the “sequelae” or injury health states with the number of injury health states expanded from 33 to 44. However, an approach for handling multiple injuries in burden estimates remains unclear.

It is common for more than one injury to occur in a single injury event and for multiple injuries to be ICD-coded for an admission. Two country-specific burden of injury studies have considered the presence of multiple injuries in their burden estimates [5], [6]. Mathers *et al*, estimated disability for only the most disabling injury under the assumption that all disability was accounted for in the weight of the most severe injury [7]. Naghavi *et al* considered the presence of up to five concurrent injuries in their approach to measuring the burden of injury and disease in Iran, under the assumption that the presence or more than five concurrent injuries was extremely rare [6]. A common disability weight was calculated using the general formula for a multiplicative model [6]. Neither study evaluated the validity of their approach to modelling injury disability burden through comparison with alternative methods.

In contrast to the injury literature, a number of studies have evaluated methods for modelling the impact of co-occurring (comorbid) health conditions on health-related quality of life (HRQL), with varying results [8], [9], [10]. Three main approaches have been evaluated; minimal, additive and multiplicative models. The “minimal” approach ignores co-existing injuries or conditions and usually the worst injury or condition “trumps” the others. With an additive or “constant decrement” model, many health conditions are included in a single regression equation and the assumption is made that the impact of each injury or health condition is the same, regardless of the presence of others [8], [10]. A multiplicative model assumes that any injury or health condition is a constant proportion of the overall health status or disability [10]. A recent study by Willis *et al* compared multiplicative, worst injury and additive approaches to modelling the impact of multiple co-existing ICD injury diagnoses on in-hospital mortality outcomes and found that the additive, multivariable approach performed best [11].

The aims of this study were to: (i) establish the 12-month disability associated with each of the GBD 2010 injury health states; and (ii) compare approaches to modelling the impact of multiple injury health states on disability.

## Methods

### Ethics statement

The Victorian State Trauma Registry and the Victorian Orthopaedic Trauma Outcomes Registry have been approved by the Human Research Ethics Committee at each participating hospital and the Monash University Human Research Ethics Committee.

### Dataset

Data from two large clinical registries were extracted for this project. The Victorian State Trauma Registry (VSTR) is a population-based trauma registry which captures data for all major trauma patients in the state of Victoria (population 5.4 million) [12], [13]. The VSTR collects data from all trauma receiving hospitals in the state. A case is defined as major trauma if it meets any of the following criteria [12], [13]: death following injury; an Injury Severity Score (ISS) >15; admission to an intensive care unit (ICU) for >24 hours; or requiring mechanical ventilation or urgent surgery (intra-thoracic, intra-abdominal, intra-cranial, or fixation of pelvic or spinal fractures). The Victorian Orthopaedic Trauma Outcomes Registry (VOTOR) is a sentinel site clinical registry which collects data about all orthopaedic trauma admissions to four hospitals in Victoria (two major trauma services, one regional trauma service and one metropolitan trauma service) [14]. Patients are eligible for inclusion if they are admitted with a new orthopaedic injury and have a length of stay greater than 24 hours. Pathological fracture admissions are excluded.

The registries use an opt-off consent process where all eligible cases are included on the registry, and patients (or their next of kin) are provided with a letter and a brochure stating the aims of the registry, the data collected, and that patients will be followed-up. The brochure provides the details for how to opt-off and the opt-off rate for both registries is less than 1%. At the follow-up interview, verbal consent to complete the interview is obtained. An opt-off consent is used due to the impracticability of informed consent, and the potential for selection bias, in the registry setting [15]. The registry protocols, including the described consent process, have been approved by the Human Research Ethics Committee of each participating hospital and Monash University. Both registries routinely capture data from the patient's hospital admission including demographic, injury event, injury diagnosis, comorbid status, treatment and in-hospital outcomes (i.e. mortality, length of stay, discharge destination, etc.).

### Inclusion criteria

All cases aged 15 years and over, and with a date of admission from 1 October 2006 to 30 June 2009 (inclusive), were extracted for analysis to correspond with the commencement of routine 12-month follow-up of VSTR patients. In-hospital deaths were excluded, as were the less than 1% of cases where the hospital did not provide ICD-10 diagnosis codes for the admission.

### Data items

For all eligible cases, demographic details, comorbid status, injury event details, in-hospital outcomes, all International Classification of Diseases 10^th^ Revision Australian modification (ICD-10-AM) diagnosis codes and the 12-month functional outcome of patients were extracted for analysis. The Charlson Comorbidity Index (CCI) was used as a measure of comorbid status and involves the weighting of 19 conditions to provide a single index of comorbid status [16], [17]. The 19 conditions were mapped to the CCI from the ICD-10-AM diagnosis codes for each admission, resulting in a weight of 1, 2, 3 or 6 [18]. If none of the ICD-10-AM diagnosis codes for the CCI conditions was allocated to the admission, a score of zero was recorded representing no comorbid conditions. The ICD-10-AM injury diagnosis codes were extracted for mapping to the GBD 2010 injury health states (http://sites.google.com/site/gbdinjuryexpertgroup/Home/discussion-3-sequelae-definition). Up to 40 individual ICD-10-AM diagnosis codes were present for each admission.

### Outcome

All adult (≥15 years) VSTR and VOTOR survivors to hospital discharge are followed-up at 6 and 12-months after injury using a standardised telephone interview to collect measures of functional and HRQL outcomes. The methodology for follow-up is published in detail elsewhere [19]. The disability outcome of interest for this project was the Glasgow Outcome Scale – Extended (GOS-E) which classifies the patient's level of function on a scale from death (GOS-E = 1) to upper good recovery (GOS-E = 8) [20]. For the purposes of this study, the GOS-E was dichotomised for analysis. The GOS-E is commonly dichotomised into a “good recovery” equivalent to a GOS-E score of 7 or 8, as this corresponds to return to work and usual social and leisure activities with no, or minimal, sequelae. The 12-month time point was used because studies have shown minimal improvement in disability outcomes after 12-months [21], [22].

### Data management and analysis

Descriptive statistics including mean and standard deviation, or median and interquartile range, were used to summarise continuous variables. Categorical variables were summarised using case counts and percentages. Multiple response tables were generated to define the distribution of GBD 2010 injury health states across the cases. Injury-specific probabilities of recovery (IPR) were generated for each injury health state as the proportion of cases with the injury health state who achieved a GOS-E score of 7 (lower good recovery) or 8 (upper good recovery) at 12-months following injury. For the worst injury model, the lowest IPR for each case was used in the model while the product of all IPRs for each case was used in the multiplicative model.

Three approaches to modelling the relationship between injury health state/s and disability were considered: a) an “additive” or multivariable model where it was assumed that the impact of each injury health state on disability was constant irrespective of the presence of other injury health states or other covariates; b) a “worst injury” or minimal approach model where only the lowest IPR was included in the model; and c) a “multiplicative” model where the product of the IPRs was included in the model, assuming that each injury health state contributed a constant proportional decrement to outcome.

A split dataset approach was used [23], with the full dataset randomly split into two equal sized samples. Models were developed on the “training” dataset and then fitted to the “test” dataset to enable internal validation of the models. The IPRs from the *training* dataset were used for all models (training and test).

All models were fitted with age, and then with and without comorbid status, as previous studies using trauma registry data have found no significant improvement in model performance from the inclusion of comorbid status over age alone using mortality as the outcome [11], [18], while studies using hospitalisations have suggested that the inclusion of comorbid status does improve the predictive performance [24]. Consistent with other trauma populations [11], [18], the prevalence of admissions with a CCI greater than one was low. Therefore, the CCI was categorised for analysis into 0 (no CCI condition), 1 (a CCI condition with a weighting of 1), 2 (CCI weighting ≥2). The models with comorbid status excluded were compared with the models with comorbid status including using a likelihood ratio test. Age was categorised into eight groups for analysis (15–24, 25–34, 35–44, 45–54, 55–64, 65–74, 75–84 and ≥85 years) as age in its continuous form was not linearly related to the log odds of recovery.

The predictive performance of the models was assessed in terms of discrimination and calibration [23], [25]. Calibration measures how accurately the models predict over the entire range and was assessed through computation of the Hosmer-Lemeshow (H–L) statistic and the construction of calibration curves. The H–L statistic partitions the observations into 10 equal groups based on their predicted probabilities (i.e. deciles of risk). Chi-squared values are then calculated as the squared differences between observed and predicted outcomes in each decile, then summed for each decile giving a chi-square value with 8 degrees of freedom [26]. Lower H–L statistics with a non-significant p-value are indicative of higher model calibration. Calibration curves plot the observed against the predicted events [27]. If there is agreement between observed and predicted values over the whole range of probabilities, the plot should show a 45° line. If the curve sits above the 45° line, this is suggestive of model under-estimation in that range of probability, and where the curve falls below the equality line suggests over-estimation of the model.

The concordance, or C-statistic, was used as a measure of model discrimination. This statistic measures the capacity of the model to discriminate between participants who experience the outcome of interest and those that do not [26], [27]. For binary logistic regression, the C-statistic is equivalent to the area under the receiver operating characteristic (ROC) curve which plots the sensitivity against 1-specificity over the range of probabilities. The area under the ROC curve (AUC) ranges from zero to one. An AUC equal to 0.5 suggests no discrimination while an AUC equal to one represents perfect discrimination. Acceptable discrimination is generally defined as an AUC ≥0.7 and <0.8, excellent discrimination as an AUC ≥0.8 and <0.9 and outstanding discrimination as an AUC ≥0.9 [26]. All analyses were performed using Stata Version 11.0 (Stata Corp, College Station, Texas). A p-value <0.05 was considered significant for all statistical tests.

## Results

### Overview of the dataset

There were 13,315 VSTR and VOTOR cases during the study period who survived to hospital discharge. Of these, 1902 (14.3%) were lost to follow-up, leaving 11,412 cases with a valid GOS-E score at 12-months. Thirty-seven of the 44 GBD 2010 health states were represented, of which 12 health states were present in less than 100 cases. For these low frequency injury health states, the case was removed if the low frequency health state was the only injury sustained by the patient (n = 75).

Overall, there were 11,337 cases in the dataset for analysis, with 5,650 randomised to the training dataset and 5,687 cases to the test dataset. The characteristics of cases in the training and test datasets were comparable (Table 1 and Table 2). A single injury health state was recorded for 46.5% of the training dataset cases and 46.0% of the test dataset cases (Table 1), with a maximum of 16 injury health states present per case. There were 1407 different patterns of injuries in the training sample and 1371 patterns in the test dataset.

**Table 1:** Characteristics of trauma registry survivors to discharge (n = 11,337).

Variable		Training dataset (n = 5650)	Test dataset (n = 5687)
**Age**	Mean (SD) years	52.8 (23.1)	52.9 (23.6)
**Gender**	n (%)		
	Male	3352 (59.3)	3381 (59.5)
	Female	2298 (40.7)	2306 (40.5)
**Cause of injury** a	n (%)		
	Low fall	2068 (36.9)	2067 (36.6)
	Motor vehicle	896 (16.0)	928 (16.4)
	High fall	686 (12.2)	656 (11.6)
	Motorcycle	579 (10.3)	585 (10.4)
	Pedal cyclist	237 (4.2)	256 (4.6)
	Pedestrian	249 (4.5)	256 (4.6)
	Struck by/collision with person	195 (3.5)	183 (3.2)
	Struck by/collision with object	157 (2.8)	169 (3.0)
	Cutting/piercing object	76 (1.4)	87 (1.5)
	Other	457 (8.2)	459 (8.1)
**Charlson Comorbidity Index Weight**	n (%)		
	None	3888 (68.8)	3853 (67.8)
	1	1281 (22.7)	1344 (23.6)
	2–6	481 (8.5)	490 (8.6)
**ICU** b **Admission**	n (%)		
	No	4740 (83.9)	4755 (83.7)
	Yes	906 (16.1)	929 (16.3)
**Hospital length of stay**	Median (IQR^c^) days	5.9 (3.0–11.1)	6.0 (3.0–11.1)
**Number of injury health states**	n (%)		
	1	2627 (46.5)	2617 (46.0)
	2	1303 (23.1)	1367 (24.0)
	3	697 (12.3)	686 (12.1)
	4	385 (6.8)	407 (7.2)
	5	258 (4.6)	255 (4.5)
	6	149 (2.6)	145 (2.6)
	>6	231 (4.1)	210 (3.6)

a Data missing for 91 cases.

b ICU - Intensive Care Unit, data missing for 7 cases.

c IQR - Interquartile range.

**Table 2:** Distribution of GBD 2010 injury health states by study sample.

Injury health state descriptor	Training dataset	Test dataset
	(n = 5650)	(n = 5687)
	n (%)^a^	n (%)^a^
Moderate/severe traumatic brain injury	1519 (27.0)	1532 (26.9)
Open wound	1345 (23.8)	1422 (25.0)
Patella/tibia/fibula fracture	1155 (20.4)	1070 (18.8)
Vertebral column fracture	1099 (19.5)	1073 (18.9)
Severe chest injury	996 (17.6)	1012 (17.8)
Radius/ulna fracture	833 (14.7)	850 (14.9)
Clavicle/scapula/humerus fracture	875 (15.5)	769 (13.5)
Neck of femur fracture	767 (13.6)	764 (13.4)
Other muscle/tendon injury	500 (8.9)	521 (9.2)
Skull fracture	466 (8.3)	487 (8.6)
Other and unspecified injuries	458 (8.1)	519 (9.1)
Facial fracture	446 (7.9)	493 (8.7)
Abdominal/pelvic organ injury	439 (7.8)	480 (8.4)
Pelvic fracture	451 (8.0)	440 (7.7)
Foot bone fracture	330 (5.8)	309 (5.4)
Femur fracture – not involving neck	294 (5.2)	299 (5.3)
Sternal/single rib fracture	281 (5.0)	280 (4.9)
Hand/wrist fracture	204 (3.6)	215 (3.8)
Knee soft tissue injury	174 (3.1)	156 (2.7)
Shoulder soft tissue injury	154 (2.7)	144 (2.5)
Eye injury	156 (2.8)	129 (2.3)
Nerve injury	124 (2.2)	110 (1.9)
Spinal cord injury – neck level	84 (1.5)	80 (1.4)
Spinal cord injury – other	47 (0.8)	71 (1.3)
Hip dislocation	58 (1.0)	59 (1.0)
Burns – minor	30 (0.5)	25 (0.4)
Poisoning	14 (0.3)	22 (0.4)
Burns ≥20% body surface area	12 (0.2)	12 (0.2)
Lower airway burns	11 (0.2)	14 (0.3)
Finger amputation	7 (0.1)	6 (0.1)
Other fracture	4 (0.1)	5 (0.1)
Amputation of one upper limb	4 (0.1)	3 (<0.1)
Burns – other serious	4 (0.1)	4 (0.1)
Amputation of one lower limb	4 (0.1)	5 (0.1)
Crush injury	2 (<0.1)	2 (<0.1)
Thumb amputation	2 (<0.1)	2 (<0.1)
Drowning/non-fatal submersion	1 (<0.1)	3 (<0.1)

a Total percentage >100% as cases can have more than one injury health state.

### Functional outcomes at 12-months

Table 3 shows the profile of GOS-E scores for the 11,337 cases at 12-months post-injury. At 12-months, 41.9% (n = 2370) of the training dataset cases, and 41.6% (n = 2367) of the test dataset cases had recovered, using a GOS-E score >6 as the definition of recovery.

**Table 3:** Functional outcomes at 12-months.

GOS-E^a^ score		Training dataset	Test dataset
		(n = 5650)	(n = 5687)
		n (%)	n (%)
1	Death	377 (6.7)	420 (7.4)
2	Vegetative state	12 (0.2)	24 (0.4)
3	Lower severe disability	691 (12.2)	681 (12.0)
4	Upper severe disability	320 (5.7)	336 (5.9)
5	Lower moderate disability	786 (13.9)	710 (12.5)
6	Upper moderate disability	1094 (19.4)	1149 (20.2)
7	Lower good recovery	901 (15.9)	957 (16.8)
8	Upper good recovery	1469 (26.0)	1410 (24.8)

a Glasgow Outcome Scale – Extended.

### Model development (training dataset)

#### Injury-specific probabilities of recovery (IPR)

The most common injury health states represented in the dataset were moderate/severe traumatic brain injury (TBI), open wounds, severe chest injuries, lower and upper limb fractures, skull fractures and organ injuries (Table 2). Twelve injury health states were recorded for fewer than 50 cases; an injury-specific probability of recovery (IPR) was not calculated as there were insufficient cases to generate a robust estimate.

Table 4 provides the IPR for each injury health state. Spinal cord injury, hip fracture, hip dislocation, and femoral fracture not involving the neck demonstrated the lowest probability of recovery and therefore the lowest IPR. The mean (SD) lowest IPR was 0.34 (0.08), and 0.20 (0.17) for the product of the IPRs, across the training dataset.

**Table 4:** Injury-specific probability of recovery (IPR) for each injury health state calculated from the training dataset (n = 5650).

Injury health state	Cases	Recovered	IPR^a^ (95% CI)
	(n)	(n)	
Spinal cord injury – neck	84	18	0.21 (0.13, 0.30)
Neck of femur fracture	767	169	0.22 (0.19, 0.25)
Hip dislocation	58	14	0.24 (0.13, 0.35)
Femur fracture – not involving neck	294	70	0.24 (0.19, 0.29)
Spinal cord injury – other	47	12	0.26 (0.13, 0.38)
Nerve injury	124	35	0.28 (0.20, 0.36)
Eye injury	156	47	0.30 (0.23, 0.37)
Pelvic fracture	451	141	0.31 (0.27, 0.36)
Other and unspecified injuries	458	153	0.33 (0.29, 0.38)
Facial fracture	446	150	0.34 (0.29, 0.38)
Open wound	1365	464	0.34 (0.32, 0.37)
Moderate/severe traumatic brain injury	1519	535	0.35 (0.33, 0.38)
Vertebral column fracture	1099	381	0.35 (0.32, 0.38)
Skull fracture	466	168	0.36 (0.32, 0.40)
Severe chest injury	996	357	0.36 (0.33, 0.39)
Knee soft tissue injury	174	62	0.36 (0.29, 0.43)
Foot bone fracture	330	118	0.36 (0.31, 0.41)
Sternal/single rib fracture	281	104	0.37 (0.31, 0.43)
Hand/wrist fracture	204	82	0.40 (0.33, 0.47)
Shoulder soft tissue injury	154	61	0.40 (0.32, 0.47)
Clavicle/scapula/humerus fracture	875	353	0.40 (0.37, 0.44)
Abdominal/pelvic organ injury	439	179	0.41 (0.36, 0.45)
Patella/tibia/fibula fracture	1155	521	0.45 (0.42, 0.48)
Other muscle/tendon injury	500	229	0.46 (0.41, 0.50)
Radius/ulna fracture	833	419	0.50 (0.47, 0.54)

a IPR; Injury probability of recovery.

#### Model performance

**Figure 1:**
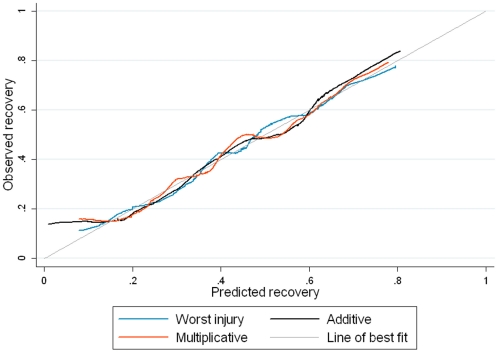
Calibration curves for models including age and comorbid status fitted in the training dataset (n = 5650). The figure is a plot the predicted versus the observed recovery in the training dataset. The 45° line represents perfect fit of the model.

Each model was fitted in the training dataset, with the results shown in Table 5. There were no missing data, and therefore all models were fitted on the full sample. All models including age were a better fit for the data than models fitted without age, and all models including comorbid status were a better for the data than models fitted with age only(Table 5). The additive and worst injury models demonstrated “acceptable” discrimination but the calibration was not adequate according to the H-L statistic (Table 5). A test of equality of the AUC was significant (X^2^_2_ = 46.0, p<0.0001) indicating that the AUC was not equal for all curves. The calibration curves were similar for all models (fitted with age and comorbid status) and largely followed the 45° line of best fit, although all models underestimated recovery at lower recovery (Figure 1).

**Table 5:** Discrimination and calibration of models in training dataset (n = 5650).

Model		Area under curve	H-L^a^ statistic	LR^b^ test
		(95% CI)	(p-value)	(p-value)
Additive	Unadjusted^c^	0.67 (0.65, 0.68)	18.63 (0.017)	
	Age	0.70 (0.69, 0.72)	23.92 (0.002)	232.58 (<0.001)
	Age and comorbidity	0.72 (0.70, 0.73)	16.50 (0.036)	98.81 (<0.001)
Worst injury	Unadjusted	0.66 (0.64, 0.67)	6.91 (0.546)	
	Age	0.69 (0.67, 0.70)	20.80 (0.008)	70.00 (<0.001)
	Age and comorbidity	0.70 (0.69, 0.72)	16.05 (0.042)	117.24 (<0.001)
Multiplicative	Unadjusted	0.61 (0.59, 0.62)	114.94 (<0.001)	
	Age	0.68 (0.67, 0.69)	36.22 (<0.001)	338.94 (<0.001)
	Age and comorbidity	0.69 (0.68, 0.71)	11.99 (0.152)	117.15 (<0.001)

a Hosmer-Lemeshow statistic.

b Likelihood ratio test.

c Model fitted without age or comorbidity.

### Model validation (test dataset)

**Figure 2:**
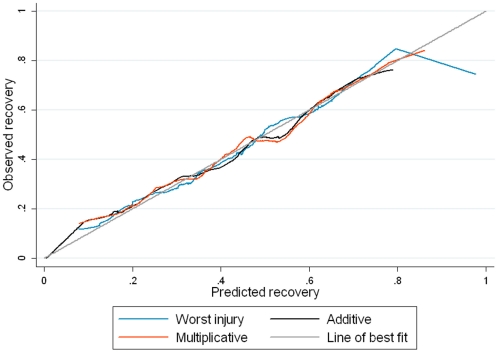
Calibration curves for models including age and comorbid status fitted in the test dataset (n = 5687). The figure is a plot the predicted versus the observed recovery in the test dataset. The 45° line represents perfect fit of the model.

The models, using the IPRs calculated from the training dataset and adjusted for age and comorbid status, were fitted in the test dataset, with the results shown in Table 6. The calibration of the additive and worst injury models was adequate according to the H-L statistic (Table 6). The discrimination of each model decreased in the test dataset, although the pattern was similar to the results from the training dataset, the additive and worst injury models achieving the highest discrimination as shown by the AUC. A test of equality of the AUC was significant (X^2^_2_ = 25.3, p<0.001) indicating that the AUC was not equal for all models. The calibration curves for each model fitted in the test dataset are shown in Figure 2. The overall calibration of the curves was relatively consistent with the training models, with all models underestimating recovery below 20%.

**Table 6:** Discrimination and calibration of models adjusted for age and comorbid status fitted in the test dataset (n = 5687).

Model	Area under curve	H-L statistic^a^
	(95% CI)	(p-value)
Additive	0.70 (0.69, 0.71)	12.77 (0.120)
Worst injury	0.70 (0.68, 0.71)	12.83 (0.118)
Multiplicative	0.68 (0.67, 0.70)	25.79 (0.001)

a Hosmer-Lemeshow statistic.

## Discussion

The aims of this study were to explore, for the first time, the GBD 2010 Study injury health states, and the performance of different approaches to modelling the relationship between these injury health states and disability at 12-months following injury. The data presented are important for guiding the methods for estimating YLD as the study provides important information about the prevalence of disability for each injury health state and is the first to evaluate the relationship between multiple injuries and disability following injury.

Using the injury health states generated for the GBD 2010 study, the prevalence of disability at 12-months post-injury across the health states was high with more than half of the cohort still affected by injury at this time point. The “worst injury”, additive and multiplicative models were developed in a training dataset and then validated using a test dataset to explore and validate different models for combining the full spectrum of injuries sustained. The results showed concordance lower than methodologically similar studies based on mortality outcomes, and no clearly superior approach to modelling these injury health states to predict recovery at 12-months following injury, although the additive and “worst injury” models showed higher concordance and discrimination than the multiplicative approach.

Numerous studies have modelled the relationship between multiple injury diagnoses and mortality following injury [11], [24], [28], [29], [30]. These studies have used routine hospital administrative data and trauma registry data, and the individual ICD diagnosis codes to model outcome. The concordance of administrative hospital data studies using ICD-10-AM diagnoses was higher, ranging from 0.78 to 0.91, although these studies used large sample sizes ranging from 186,835 admissions to more than 500,000 admissions [24], [30]. A study using Australian trauma registry data compared multiplicative, additive and “worst injury” models for predicting mortality developed and validated in samples of similar size to the current study (>5000) found concordance ranging from 0.80 to 0.90 [11].

In comparison, the concordances observed in the test dataset in the current study did not exceed 0.70, which equates to a 70% chance that given two patients, one who will recover and one who will continue to have disability at 12-months, the model will assign a higher probability of recovery to the patient who recovers. Only the additive and “worst injury” models demonstrated acceptable calibration in the test dataset, suggesting problems with goodness-of-fit for the multiplicative approach.

The lower concordance and variation from perfect fit of the calibration curves could suggest that recovery after injury is more difficult to predict than mortality and/or reflect the injury health states evaluated. Cohort studies have found additional factors not included in the current models, such as level of education, marital status, socioeconomic status, compensation status and injury severity, to be important predictors of long term outcome after injury [22], [31], [32], [33], [34], [35], [36]. It is likely that the inclusion of additional factors would increase the predictive performance of the models. However, while the VSTR and VOTOR collect many of these factors routinely, they are not considered by the GBD Study in the calculation of the YLD component of the DALYs for injury and were therefore excluded from this study.

Most studies of mortality following injury have used individual ICD diagnosis codes to represent injury conditions in models. In the current study, we modelled ICD-coded data after collapsing the more than 1200 ICD-10 injury diagnosis codes into 44 GBD 2010 injury health states. Many of the injury health states combine a number of injury diagnoses, potentially combining injuries with different probabilities of recovery and duration of disability into a single group. Evidence of this heterogeneity can be seen in Table 4. The most specific injury health states performed as expected. For example, spinal cord injury at the neck level was associated with the lowest probability of recovery, and fractures to the femur (neck or other) also demonstrated low probabilities of recovery, which is consistent with clinical and cohort studies. Similarly, the probability of recovery for patients with radius and ulna (forearm) fractures was highest, reflecting the usually low severity and short recovery time of this injury, and the fact that cases occur most commonly in isolation. An exception is the “moderate and severe traumatic brain injury” health state, which showed a higher probability of recovery than expected, given that severe brain injury commonly leads to marked and permanent disability. Where injuries with different probabilities of recovery are bundled together into a single health state, the overall probability of recovery will be heavily influenced by the more prevalent condition. In this instance, moderate traumatic brain injury is more common than severe traumatic brain injury, potentially explaining the higher than expected IPR for this health state. While grouping ICD codes into the GBD 2010 injury health states certainly increases heterogeneity, it should be acknowledged that the ICD-10-AM classification itself cannot be expected to achieve complete homogeneity in the groups of cases that it distinguishes, further challenging the development of optimal injury health states.

Overall, more than half of the study sample had sustained more than one injury health state, with 7% sustaining more than five, an occurrence considered “extremely rare” by the authors of the Iranian burden of disease and injury study[6]. The prevalence of multiple injuries reflects the inclusion criteria of the registries, particularly the VSTR, but highlights the need to develop an approach for consideration of multiple injuries in burden estimates. Previous burden of injury studies have used a multiplicative approach [6] or a “worst injury” approach [5], but previous studies have not compared different approaches. In the current study, the additive model performed better for modelling the presence of multiple injuries than the multiplicative model, consistent with the mortality study of Willis *et al* [11], but was similar in performance to the “worst injury” model. The findings support the approach used by Mathers *et al* and suggest that an additive model performs better than multiplicative approaches when combining all injuries sustained.

This is the first study investigating modelling approaches to disability after injury and limitations of the study require acknowledgement. The data were drawn from trauma registries which focus on severe and orthopaedic injury cases. Consequently, some GBD injury health states were not represented at all in the data or were represented by too few cases to generate a reliable estimate of the probability of recovery. Additionally, injury health states involving combined more than one injury type were likely to be over-represented by the more severe injury in the injury health state. For example, moderate to severe TBI would likely include a higher proportion of severe head injured patients than a more general hospital discharge dataset due to the inclusion criteria for the VSTR. The implications of the case-mix on the generalisability of the study findings are not clear as comparable disability datasets are not available. However, given that hospital discharge datasets would likely contain a wider distribution of injury severities, and greater heterogeneity in disability outcomes, the potential for reduced model fit is possible.

The follow-up rate at 12-months was 86% of all registered patients. Whether the disability outcomes of the patients lost to follow-up differed to the respondents is not known. It should be noted that follow-up commenced for nearly all patients who survived to discharge, because only about 1% of patients had opted-out of the registers. In contrast, studies based on an opt-in consent process typically can commence follow-up on only about half of the discharged patients, with much greater potential for bias [15], [19]. The study involved internal model validation, with the test dataset drawn from the same population as the training dataset, an approach likely to give optimistic results in the test dataset due to the similarity of the datasets [23]. External validation is desirable.

Overall, the majority of patients survived their injuries but were not fully recovered 12 months after onset. The evident potential for injury patients developing persistent disability highlights the importance of improving methods for estimating of the burden of non-fatal injury, and for applying them. This study was a first attempt to assess the relationship between the 2010 GBD injury health states and long term disability, including the investigation of modelling different methods of handling multiple injuries. The results show that the additive and “worst injury” models performed better than the multiplicative model, although concordance did not exceed 0.70 for any model. Factors likely to have contributed to the relatively poor fit were heterogeneity for the study outcome in at least some of the GBD 2010 injury health states, and use of models that did not include certain known predictors of the outcome (in order to replicate GBD methods). The next steps will be to investigate improved classification of injury health states, the handling of post-discharge and longer term mortality in burden estimates, and investigation of additional outcomes such as health-related quality of life. The burden based on GBD 2010 Disability Weights, which had not been released at the time of writing, will be compared with burden based on prospectively measured outcomes.
